# Ion Selective Electrode (ISE) Method for Determination of Total Fluorine and Total Organic Fluorine in Packaging Substrates

**DOI:** 10.3390/mps6010010

**Published:** 2023-01-18

**Authors:** Ma Cristine Concepcion D. Ignacio, Greg W. Curtzwiler, Mark R. Early, Katie M. Updegraff, Keith L. Vorst

**Affiliations:** 1 Department of Agricultural and Biosystems Engineering, Iowa State University, Ames, IA 50011, USA; 2 Department of Food Science and Human Nutrition, Iowa State University, Ames, IA 50011, USA

**Keywords:** per- and polyfluoroalkyl substances (PFAS), food packaging materials, total fluorine, total organic fluorine, ion selective electrode (ISE)

## Abstract

Various testing methods and techniques have been used to identify and quantify per- and polyfluoroalkyl substances (PFAS) in food packaging. A common indirect measurement of PFAS is total fluorine (TF) and total organic fluorine (TOF). These methods are critical in rapidly screening food packaging materials for the >9000 PFAS and are often globally used for regulatory limits. However, this destructive approach requires careful sample preparation, combustion, and the analysis of the solution by a fluoride-specific electrode. The method described herein is a cost-effective, rapid, quantitative, and externally validated initial screening of packaging materials for fluoro-chemistry. This study presents validated protocols for measuring TF and TOF in packaging substrates using oxygen combustion sample preparation coupled with fluoride ion-selective electrode (F-ISE); the materials and required equipment are provided, and the step-by-step procedure from sample preparation to the analysis are described, including critical steps to minimize contamination and interferences during sample preparation.

## 1. Introduction

Total fluorine measurements in consumer packaging are often used to rapidly screen food contact materials (FCM) for the presence of per- and polyfluoroalkyl substances (PFAS). These chemical compounds have been extensively used in consumer packaging because of their resistance to fat, oil, grease, high temperature, water, and degradation, especially in convenience and retail packaging applications [1,2,3]. However, concerns with PFAS and fluoro-chemistries in FCMs are continually increasing because of documented harmful effects on humans and the environment [4]. Some PFAS are persistent, bio accumulative, and migrate from food packaging into food, landfills, and the environment [5]. Moreover, studies have shown that PFAS are associated with adverse human health effects, such as cancer, immunotoxicity, reproductive harm, and thyroid disease. Thus, exposure to PFAS should be minimized [2,4,6].

Many countries have established regulations for using these compounds in FCMs to limit potential harm to human and environmental health. Certain types of PFAS are currently banned in the US and some European countries for food contact packaging applications [2,7]. However, some PFAS are still authorized by the FDA for limited use in cookware, food packaging, and food processing equipment. A threshold of 100 parts per million (micrograms per gram, grams per kilogram, microgram per milliliter, or gram per liter) of total fluorine (as fluoride ion) has been adopted by many brand owners and retailers as a concentration that is indicative of intentionally added PFAS treatments or the results of environmental contamination, such as process water, recycled materials, or unknown processing aids. This limit is often selected to harmonize with brand owners and manufacturers with compostability certifications, such as the Biodegradable Products Institute [8,9]. However, our recent work demonstrated that the PFAS concentration that increases water and oil resistance can range from 40 to >1200 µg/g depending on the number of fluorinated carbons [7]. Recent changes in 2020 include the implementation of bans on the use of all PFAS compounds in paper and paper board FCMs and introduced an indicator threshold value of 20 micrograms of organic fluorine per gram (ppm) of paper to help the industry assess intentionally added PFAS [10].

Several analytical approaches, both destructive and non-destructive, have emerged to evaluate total fluorine in FCMs, including combustion ion chromatography (CIC), particle-induced γ-ray emission spectroscopy (PIGE), instrumental neutron activation analysis (INAA), and combustion ion-specific electrode (ISE) [2,3,10]; all methods have been demonstrated to produce reliable measurements [3]. However, these methods vary by several factors, including sample preparation (destructive vs. non-destructive), detection limits, accuracy and precision, cost, equipment needed, and training requirements for personnel. The combustion ISE method presented in this study is the simplest and most inexpensive analytical technique among the aforementioned methods. It also provides accurate results when the correct precautions are taken, limiting cross contamination during sample preparation, and the ISE probe is frequently validated and calibrated. Ion selective electrodes are used throughout industry and academia for a variety of analyses, including groundwater monitoring, biomedical laboratories, clinical and environmental analysis, physiology, and process control [11]. Moreover, several studies have shown the use of ISE on the determination of total fluorine on different matrices, such as foams, fuels, lubricants, and organic materials [12,13,14]. Additionally, the successful fabrication of ISE from a polyurethane membrane for lead analysis was reported in several research studies [15,16,17]. An overview of common analytical techniques for the evaluation of TF/TOF and PFAS [18] is described in Table 1.

The method described herein utilizes fluoride ion-selective electrodes (F-ISE) combined with oxygen combustion sample preparation to measure the total fluorine and total organic fluorine of food contact materials. This destructive method involving sample preparation, combustion, and the analysis of the solution by a fluoride-ion specific electrode is a cost effective, rapid, and quantitative tool for the initial screening of packaging materials for fluoro-chemistry. F-ISE measurements are generated under the principle of direct potentiometric measurement of the voltage of a galvanic cell, which is measured with two reference electrodes positioned in the respective aqueous phases [19]. The fluoride ion probe consists of an inner reference electrode plus a membrane that provides the interface between the sample solution and the ISE. The output potential is proportional to the fluoride ion concentration in the solution and the activity of the fluoride ions on each side of the membrane.

Establishing a validated analytical technique for total fluorine and total organic fluorine measurements is critical to ensuring the consumer and environmental safety of all FCMs. Compared to other methods, F-ISE is a cost-effective approach that suppliers and manufacturers can adopt in quality control departments without using sophisticated analytical equipment and reduced training to perform the analysis.

## 2. Experimental Design

**Figure 1:**
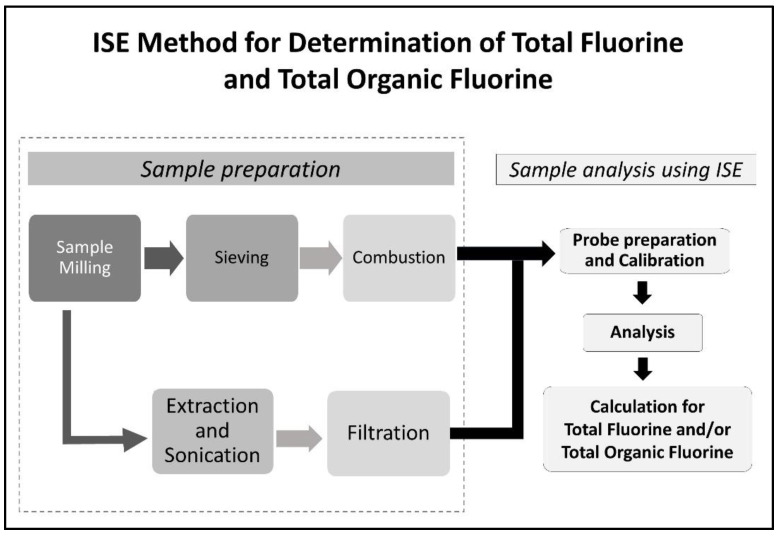
ISE total fluorine and total organic fluorine measurement process chart.

Figure 1 describes the overall process of analyzing the total fluorine and total organic fluorine of food contact materials using the oxygen combustion/F-ISE detection method. Samples should be free from any food materials and impurities to ensure accurate calculations and that the fluoride response is only attributed to the packaging material. After milling, samples will be screened using a 425 μm sieve and oxygen combustion vessel to control particle size in the gelatin capsule prior to combustion. The total organic fluorine analysis is an appropriate “next step” for samples with a high total fluorine level (greater than 20 ppm according to the Danish regulatory [10]) because it eliminates inorganic fluorine interference.

### 2.1. Materials

The materials used in this protocol include:

1. Beaker, 250 mL (Pyrex^®^, Greencastle, PA, USA)
2. Ceramic filter (Fisherbrand™, Chelmsford, MA, USA)
3. Distilled water
4. Distilled water bath
5. Erlenmeyer flask, 250 (Pyrex^®^, Greencastle, PA, USA)
6. Optimum results fluoride electrode filling solution (Thermo Fisher Scientific, Chelmsford, MA, USA; Cat. no.: 900061)
7. Fluoride with TISAB II standard (Thermo Fisher Scientific, Chelmsford, MA, USA), 10 ppm (Cat. No. 040908), 5 ppm (Cat. No. F40905), 2 ppm (Cat. No. 040907), 1 ppm (Cat. No. 040906), 0.5 ppm (Cat. No. F40904)
8. Fuse ignition wire for oxygen combustion vessel (Parr Instrument Company, Moline, IL, USA)
9. Glass sample vials
10. Gelatin capsule, Size 00, 0.9 mL capacity (Parr Instrument Company, Moline, IL, USA)
11. Liquid nitrogen, 99.5%
12. Oxygen, 99.5%
13. Sodium bicarbonate (Thermo Fisher Scientific, Chelmsford, MA, USA; Cas No. 144-55-8)
14. Sodium carbonate anhydrous (Thermo Fisher Scientific, Chelmsford, MA, USA; Cas No. 497-19-8)
15. Total ionic strength adjustment buffer, TISAB II (LabChem, Zellenople, PA, USA; Cat. no.: LC2621305)
16. Volumetric flask, 50 mL (Pyrex^®^, Greencastle, PA, USA)
17. Water, ACS reagent grade, ASTM Type II (Ricca Chemical Company, Arlington, TX, USA; Cat. no.: 9152-5)
18. Sampling bag, ethylene oxide (ETO) sterilized polyethylene bag with wire
19. Whatman™ filter paper No. 4 (Cytiva, Marlboro, MA, USA; Cat. no.: 1441-125)

### 2.2. Equipment

The equipment used in this protocol includes:

- Basic mill, A11 basic analytical mill, S001 Model with a 2905200440SS Cutting Blade (IKA Works Inc., Wilmington, NC, USA) or equivalent
- Combustion capsules (crucible), 43AS (IKA Works Inc., Wilmington, NC, USA) or equivalent
- Digital weighing balance, SI-234 summit series analytical balance, 230 g × 0.1 Mg, 115 V, with internal calibration (Denver Instrument Company, Arvada, CO, USA)
- Ignition unit, 2901EB model, 115 V (Parr Instrument Company, Moline, IL, USA) or equivalent
- Laboratory test sieve, 425-micron, stainless steel mesh and frame (Endecotts Ltd., London, UK)
- Tip sonicator, Fisherbrand™ Model FB505 (Fisherbrand™, Chelmsford, MA, USA) or equivalent
- Fisherbrand™ tip probe, 6 mm diameter (Fisherbrand™, Chelmsford, MA, USA) or equivalent
- Orion fluoride electrode, 9609BNWP (Thermo Fisher Scientific, Chelmsford, MA, USA; Cat. No. 900100) or equivalent
- Orion Dual Star pH, ISE, mV, ORP, and temperature dual channel benchtop meter (Thermo Fisher Scientific, Chelmsford, MA, USA) or equivalent
- Oxygen combustion vessel, 118 Series (Parr Instrument Company, Moline, IL, USA) or equivalent
- Oxygen tank
- Spare grinding chamber, A11.5 (IKA Works Inc., Wilmington, NC, USA) or equivalent
- Vortex mixer, 120 V (Thermo Fisher Scientific, Chelmsford, MA, USA; Cat no.: 02215365) or equivalent

## 3. Procedure

### 3.1. Total Fluorine Measurement

#### 3.1.1. Sample Preparation

- Milling and Sieving

1. Milling Sample

**Figure 2:**
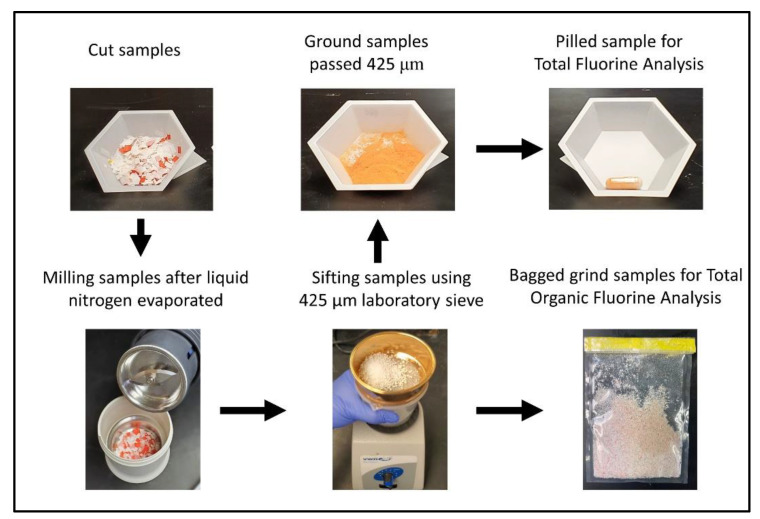
Sample preparation process for measuring total fluorine/total organic fluorine.

Cut samples into ½ inch squares and place into an A 11.5 Spare grinding chamber until the metal section is about 3/4 full. Fill the chamber with liquid nitrogen so that the sample is fully covered, making sure that the metal aspect of the chamber is the only partly exposed to the liquid nitrogen. When the liquid nitrogen has evaporated fully, mill the cut samples using the IKA A11 basic analytical mill for no longer than 60 s (Figure 2). Repeat this process until the milled sample is small enough to pass through a 425 μm sieve. It may be necessary to repeat this process 3–5 times depending on the substrate.

2. Sieving and Preparing Pilled Sample

Sift milled samples through a 425 μm stainless steel test sieve. After sieving, weigh a 100 to 400 mg sample into a tared gelatin capsule and, if needed, add no more than 1.1 g of any combustion aid (e.g., benzoic acid, gelatin, firing oil, starch) or until the gelatin capsule is full. When testing paper samples, it may be necessary to compact the sample in the gelatin capsule using a metal or glass rod to achieve the minimum mass required for combustion. Record the mass of the sample and place the capsule in a glass sample vial with a label containing the sample name and weight. Prepare three (3) gelatin capsules (pilled samples) for each sample to be tested. Additional samples can be made in the event of a failed combustion (defined later).

Place the milled samples that did not pass the 425 μm sieve in an ethylene oxide (ETO) sterilized polyethylene bag with the label name for total organic fluorine analysis.

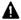


**CRITICAL STEP**

It is important to continue the cooling and milling process until the samples are small enough to pass through a 425 μm sieve. The milling time could take longer depending on the food packaging material to be tested.

- Combustion

1. 1. Attaching pilled sample

**Figure 3:**
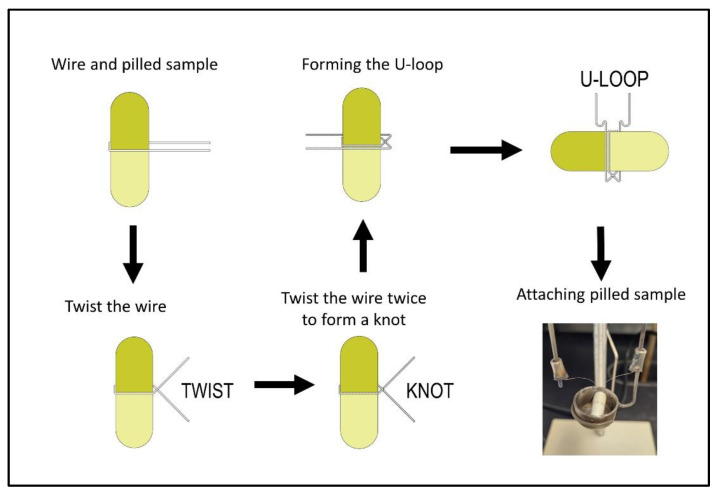
Preparing and attaching pilled sample into the combustion cup.

Next, prepare the pilled sample for combustion by placing the combustion vessel with the fuse on a support stand, as shown in Figure 3. Unravel approximately 25 cm of the ignition wire and wrap it around the pilled sample, forming a “U” shape around the pill (Figure 3). Grasp the ignition wires tightly with one hand and twist the pilled sample so that a tight knot forms in the ignition wire without damaging the gelatin capsule. Carefully place the pilled sample with the ignition wire into the combustion cup and then fasten the wire between the two electrodes. Pinch the ignition wire around the capsule, so that the wire is in direct contact with the capsule.

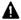


**CRITICAL STEP**

When wrapping the capsule with ignition wire, ensure that the wire is not touching any part of the crucible.

2. Preparing the combustion vessel

First, prepare the absorption solution by mixing 2.54 g of sodium carbonate and 2.52 g of sodium bicarbonate with 1.0 L of ACS reagent grade water. Store in the refrigerator.

**Figure 4:**
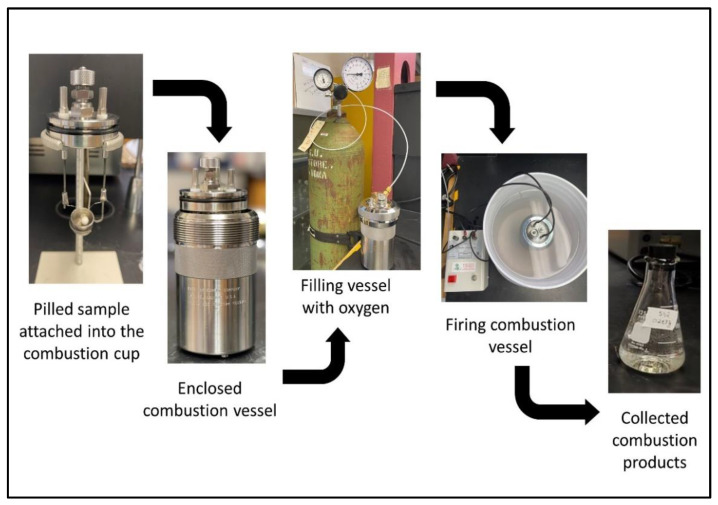
Combustion process procedure.

Add 10 mL of absorption solution to the lower half of the combustion vessel and place the combustion vessel head in the cylinder (Figure 4). Carefully push down the combustion vessel head as far as possible into the combustion vessel with the gas release valve open. Then, close the gas release valve and place the screw cap on the cylinder and turn it down firmly by hand to a solid stop to close the combustion vessel [20].

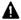


**CRITICAL STEP**

Inspect the sealing ring before pushing down the combustion vessel head into the cylinder. Wet the sealing ring with water to help the oxygen combustion vessel head slide freely into the cylinder [20]. All seals and O-rings must be replaced after 500 firings, every six months, or if the seal is visibly damaged or cannot provide a proper seal.

3. Firing the combustion vessel

Slide the oxygen filling hose connector onto the inlet valve body and push it down as far as possible (Figure 4). Slowly open the filling connection control valve while watching the pressure gage as the vessel pressure rises to the targeted pressure of 30 atm, then close the control valve on the oxygen tank. Next, slowly open the gas release valve to purge residual air from the combustion vessel. Note: rapid pressure changes in the combustion vessel can influence the position of the gelatin capsule in the crucible. Close the valve when the pressure reaches 5 atm. Then, slowly open the filling connection valve on the oxygen source until it reaches 30 atm and close it. Lastly, release the residual pressure in the filling hose by pushing downward on the lever attached to the relief valve (note: gauge should read zero). Remove the filling hose from the oxygen combustion vessel [20].

Connect one of the lead wires (10 cm) from the ignition unit to one of the electrodes of the oxygen combustion vessel using the banana plug. Then connect the other wire (common) from the ignition unit to one of the electrodes of the oxygen combustion vessel using the banana plug [20]. Carefully lower the combustion vessel (connected to the wires) into the center of the water bucket.

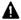


**CRITICAL STEP**

The combustion vessel should be completely submerged in water during firing. Check for the presence of continuous bubbling from the combustion vessel. If bubbles are continuously present, do not fire the vessel, as the vessel is not properly sealed [20]. Remove the combustion vessel from the bucket and start over. Do not place any body parts over the combustion vessel when firing. Stand away from the combustion vessel during ignition.

Plug the ignition unit into an electrical receptacle. Fire the charge by pressing the firing button on the ignition unit until the light on the ignition unit turns off (press for 5 s). Unplug the ignition unit from the electrical receptacle.

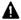


**CRITICAL STEP**

If the light continues to glow while the button is depressed, there is either a short circuit or the fuse is not properly set. Do not handle the combustion vessel for at least 6 min after attempting to fire.

4. Collecting combustion products

Allow the combustion vessel to remain in the water bath for at least 6 min after firing, then remove it from the water bath. Wipe the exterior of the combustion vessel with a paper towel. Open the gas release valve slightly to slowly release residual gas over at least one minute to avoid entrainment losses. Then remove the screw cap and carefully pull the combustion vessel head out of the combustion vessel [20]. Place the combustion vessel head on the stand. Examine the combustion vessel for evidence of incomplete combustion, such as solid material in the absorption solution. The absorption solution should be clear and absent of any particulates.

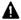


**CRITICAL STEP**

**Figure 5:**
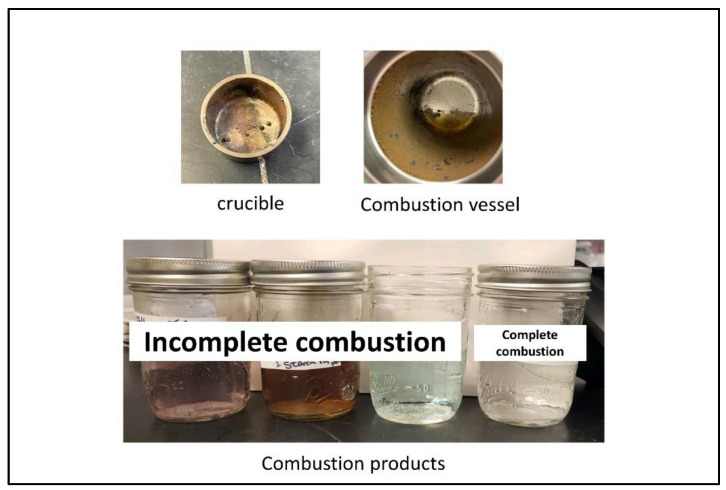
Sample of incomplete combustion.

Incomplete combustion (Figure 5) means that the test is faulty, and you need to start over. A small amount of soot is expected, but if any uncombusted material is still present in the crucible it should be considered a failure.

Transfer the combustion products to a 50 mL volumetric flask. Measure 35 mL ACS reagent grade water in a clean 50 mL graduated cylinder and slowly pour it down the walls of the combustion vessel and into the combustion cup to rinse all combustion products into the solution in the combustion vessel. Then, transfer the rinse water into the 50 mL volumetric flask containing the original combustion vessel contents and dilute to the volumetric line with ACS water.

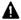


**CRITICAL STEP**

Rinse the combustion vessel inside, including the combustion cup, with ACS reagent water to recover all combustion products potentially adsorbed on the combustion vessel walls.

Transfer the contents of the 50 mL volumetric flask into a 250 mL Erlenmeyer flask capped with a rubber stopper. Then, place the Erlenmeyer flask on a vortex for one minute to degas the solution. Measure 50 mL of TISAB II solution using a 50 mL volumetric flask and add it to the Erlenmeyer flask with the diluted combustion product (10 mL carbonate solution plus 35 mL ACS reagent grade water used to rinse the combustion vessel). The 250 Erlenmeyer flask should now contain a total of 100 mL of solution—10 mL of sodium carbonate solution used in combustion vessel, 35 mL of ACS reagent grade water used to rinse the vessel after combustion, and 50 mL of TISAB II. Label the 250 mL Erlenmeyer flask with a sample description and the starting capsule mass.

#### 3.1.2. Sample Analysis Electrode preparation

**Figure 6:**
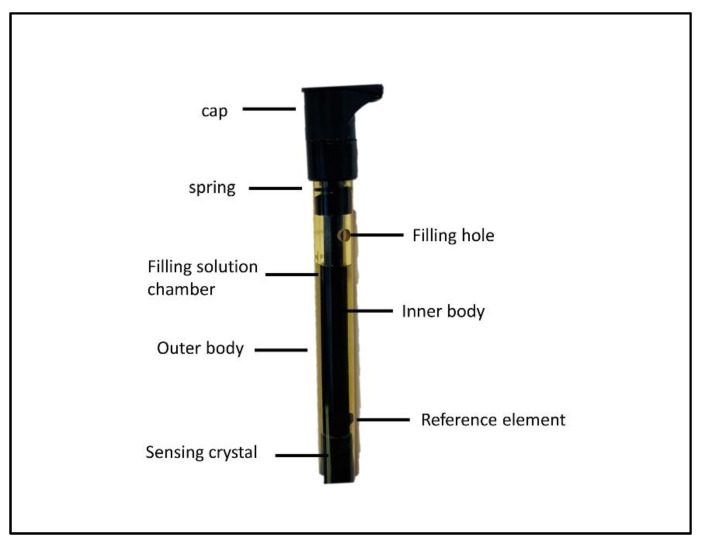
Fluoride selective electrode probe.

Fill the fluoride selective electrode (Figure 6) with the *“A Optimum Results* filling solution”. This filling solution minimizes junction potential issues and fluoride contamination in the sample. Lift the flip spout on the filling solution bottle to a vertical position, then insert the spout into the filling hole on the outer body of the electrode and add a small amount of filling solution to the reference chamber. Invert the electrode to moisten the top O-ring and then return the electrode to the upright position.

Hold the electrode body with one hand and use your thumb to push down on the electrode cap to allow a few drops of filling solution to drain out of the electrode. Release the electrode cap. If the sleeve does not return to its original position, check if the O-ring is moist and repeat steps 2–4 until the sleeve returns to its original position. Add the filling solution to the electrode up to the filling hole [21].

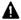


**CRITICAL STEP**

Add filling solution each day before using the electrode. The filling solution level should be at least one inch above the level of the sample in the beaker to ensure a proper flow rate. The fill hole should always be open when taking measurements.

- Calibration check of electrode

If the electrode has been stored dry, prepare the electrode as described in the “Electrode Preparation” section of the operating. Ensure that the electrode is connected to the meter. Set the meter to the mV mode by pressing “Mode” and finding the mV setting. The following procedures are two options for performing a calibration and linearity check on the electrode [21].

1. Check Electrode Linearity (Slope)

Measure 50 mL of distilled water and 50 mL of TISAB II (with CDTA) each in a separate 50 mL volumetric flask and add them into a 150 mL beaker, then stir the solution thoroughly. Rinse the electrode with distilled water and place the electrode into the prepared distilled water and TISAB II solution. Pipette 1 mL of the standard into the beaker and stir thoroughly. The standard can either be a 0.1M sodium fluoride or 100 ppm fluoride ion (F^−^) (100 µg F^−^ per mL water) solution with a sodium counterion. Lower the electrode into the beaker with the solution with a standard. Record the electrode potential in millivolts (mV) when a stable reading is displayed. Pipette 10 mL of the same standard into the same beaker, stir thoroughly, and measure for the second time [21].

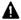


**CRITICAL STEP**

There should be a 54 to 60 mV difference between the two millivolt readings when the solution temperature is between 20 to 25 °C. If the millivolt potential is not within this range, refer to the “Troubleshooting” section of the electrode and meter operations manual [21].

2. Creating External Calibration Curve

Prepare eight fluoride standards with TISAB II (0.1, 1, 10, 25, 50, 100, 500, and 1000 ppm) (Table 2). If stored in the refrigerator, allow them to reach room temperature before use for precise measurements. Confirm that the electrode is connected to the meter and set the meter to the ISE mode by pressing “Mode” and finding the mV setting. Begin the calibration by rinsing the probe with ACS reagent grade water into a discard beaker, wiping with a low lint wipe, then inserting the ISE probe into the 0.1 ppm standard. Allow the reading to stabilize and record the mV reading. Repeat the process for 1 ppm, 10 ppm, 25 ppm, 50 ppm, 100 ppm, 500 ppm, and 1000 ppm standards.

3. Analysis

**Figure 7:**
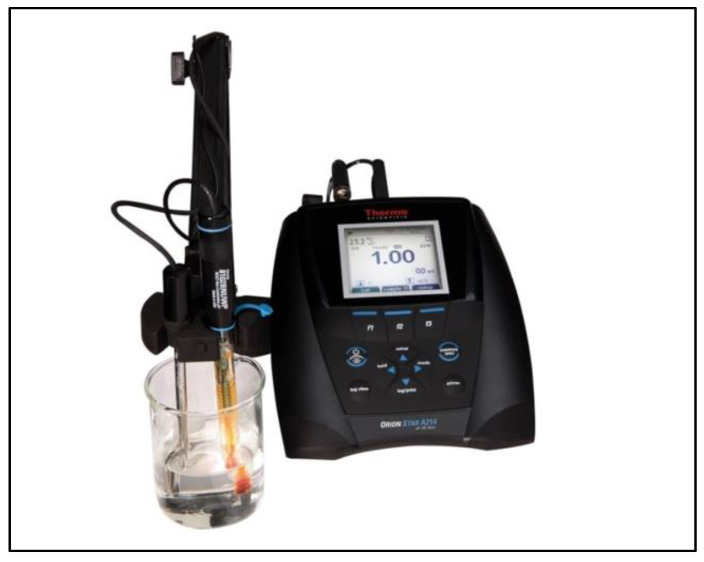
Sample analysis using F-ISE.

The total fluorine (as fluoride ion) concentration of solutions is measured using an Orion fluoride electrode and Orion Dual Star pH, ISE, mV, ORP, and temperature dual channel benchtop meter or equivalent. Always rinse the ISE with deionized water and blot dry with a low lint wipe before analyzing samples. Transfer the 100 mL of unknown solution from its Erlenmeyer flask to a 250 mL beaker. Lower the electrode into the unknown solution, as shown in Figure 7. Ensure that the temperature probe and stir rod are on and submerged into the beaker and that the filling solution in the electrode is above the solution level. When the reading is stable, record the mV value. Rinse each apparatus with ACS reagent grade water and blot dry between each sample measured.

4. Calculation

a. Determination of Calibration Slope

Using Microsoft Excel or equivalent, plot the natural log of the eight fluoride ion concentration standards (0.1, 1, 10, 25, 50, 100, 500, and 1000 ppm (µg/mL)) against their corresponding measured mV values to create an 8-point calibration curve. Fit the data points with a linear least-squares line and obtain the slope-intercept equation. The slope and y-intercept will be used to convert the millivolts measured in the post-combustion solution to ppm of solution (µg/mL).

b. Converting millivolt reading to Fluoride concentration in ppm

After measuring the millivolt value, use the slope-intercept equation (Equation (1)) to convert to parts per million, where the y variable (y) is the millivolt value, the slope (m) is the slope obtained by the 8-point calibration curve, the x variable (x) is the unknown parts per million value in natural logarithm, and the y-intercept (b) is the y-intercept obtained by the 8-point calibration curve.

(1) y=m(x)+b

Substitute the calculated x value into the inverse natural log (exponential function, e^x^). The resulting value will be ppm in solution (µg/mL).

c. Conversion from ppm of solution (µg/mL) to ppm of sample (mg/kg)

To convert the results from the ppm of solution (µg/mL) to ppm of sample (mg/kg), use Equation (2). The volume before TISAB addition should be 50 mL if using the above procedure.

(2) $$\mathrm{ppm}\;\left(\;\frac{{µg\;F}^{-}}{\mathrm{ml}\;\mathrm{of}\;\mathrm{solution}}\;\right)\times \;\mathrm{volume}\;\mathrm{before}\;\mathrm{TISAB}\;\mathrm{addition}\;\left(\mathrm{mL}\right)\times \left(\frac{1\;\mathrm{mg}}{1000\;µg}\right)={\mathrm{mg}\;F}^{-}$$

Subtract your blank value (mg) from each of your unknown values (mg). This will eliminate any [F^−^] reading from the blank capsule. Divide unknown samples by the starting mass in kg. The final ppm will be the ppm of the sample (mg of fluorine measured/kg of sample combusted).

### 3.2. Cleaning Oxygen Combustion Vessel and Other Materials Used in Milling

Clean the oxygen combustion vessel before use, in between each combustion, and after use. Using an Alconox solution, cover areas with soot and sit for about 5 min. Thoroughly scrub the crucible, vessel walls and top with water and Alconox solution. If any soot remains, gently scrub the area with a heavy-duty scouring pad. Rinse with methanol and ACS water, ensuring that water comes in contact with all surfaces. Rinse with a small amount of methanol and pour into an appropriate waste container. Place the vessel upside down on a paper towel and let it air dry.

Wash the spatulas, analytical mill blades, sieves, test tubes, ceramic filters, and collection containers with soapy water and rinse thoroughly with methanol between samples. Remove any residual particles attached to the scissors, analytical mill base, and vortex with compressed air and wipe down with methanol and a lint-free wipe.

### 3.3. Total Organic Fluorine Measurement

#### 3.3.1. Sample Preparation

**Figure 8:**
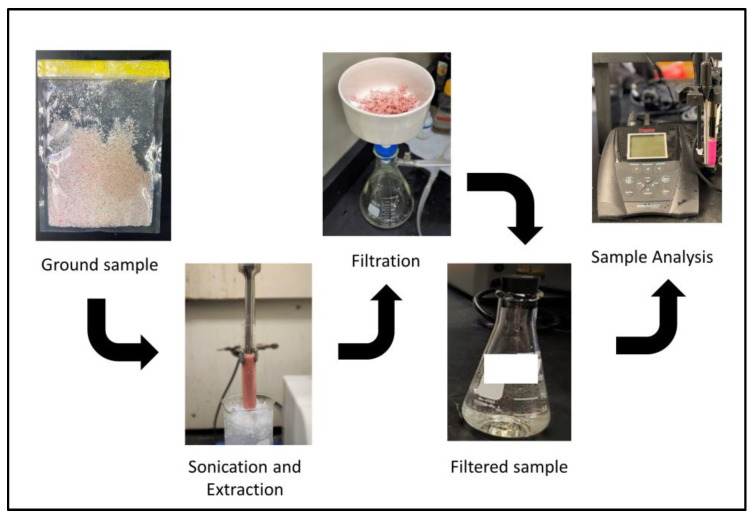
Total organic fluorine test procedure.

Measure three grams of finely milled samples and place them in 50 mL test tube with a screw cap and a mouth wide enough to fit the tip sonicator. Add 30 mL of ACS reagent grade water into the milled samples. Then place a tip sonicator in the test tube and run for 60 s at 20 kHz (Figure 8). Rinse the tip sonicator with DI water and blot dry between each sample.

Filter the sample using a ceramic filter and number 4 filter paper that has been qualified to not contain detectable concentrations of inorganic fluoride. Dilute 10 or 20 mL of the filtered solution in a 1:1 ratio with TISAB II.

#### 3.3.2. Sample Analysis

The inorganic fluoride in the extracted samples will be quantified using an external 8-point calibration curve consisting of 0.1, 1, 10, 25, 50, 100, 500, and 1000 ppm (μg/mL) standards (external calibration method). Measure the extracted total inorganic fluorine of the diluted solution using an Orion fluoride electrode and Orion Dual Star pH, ISE, mV, ORP, and temperature dual channel benchtop meter. The measured value indicates the amount of inorganic fluorine extracted from the sample. This value will be subtracted from the initial total fluorine value, yielding a total organic fluorine value.

### 3.4. Recommended Templates

Table 3 shows the sample preparation data sheet that can be used for total fluorine analysis while Table 4 demonstrates calculating and reporting the fluoride ion concentration of unknown samples in ppm (μg/mL or mg/kg).

## 4. Results

### 4.1. Limit of Detection

**Figure 9:**
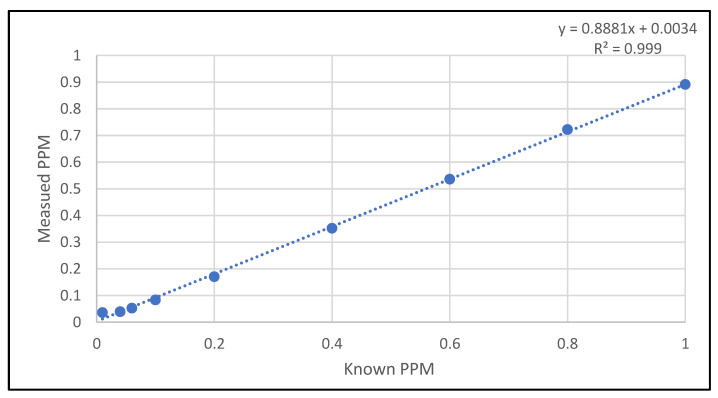
Plotting known total fluorine concentration against measured values.

The method’s detection limit is determined by testing the F-ISE probe using standards of varying concentrations and plotting against the measured values (Figure 9) following the EPA procedures on detection and quantification under the Clean Water Act analytical methods [22]. One standard solution was prepared for each concentration and measured the mV value three times. The average mV values are then calculated based on a calibration curve to calculate the total fluorine concentration (ppm). The instrument limit of detection (ILOD) is identified to be 0.1 ppm, while the method limit of detection (MLOD) was calculated as 20 ppm using Equation (3).

(3) $$\mathrm{MLOD}\;\left(\frac{\mathrm{mg}}{\mathrm{kg}};\;\mathrm{ppm}\right)=\;\mathrm{ILOD}\;\left(\frac{\mu g}{\mathrm{mL}}\right)\times \frac{\mathrm{volume}\;\mathrm{of}\;\mathrm{total}\;\mathrm{solution}\;\left(\mathrm{mL}\right)\;}{\mathrm{sample}\;\mathrm{mass}\;\left(g\right)}$$

### 4.2. Repeatability

Three types of food packaging substrates were used to test the repeatability of the method. Five measurements were collected for each sample prepared from a single milling and sieving. The mean, standard deviation, and coefficient of variation (%CV) were calculated and shown in Table 5. Results showed that all measurements have CV < 10%.

### 4.3. External Laboratory Validation

An external laboratory conducted the validation of the F-ISE method described above. The accuracy was evaluated by spiking samples pre-extracted with sodium fluoride at varying concentrations [23]. Results demonstrated (Table 6) that the method is accurate for percent recoveries from 80% to 120%.

### 4.4. Cross Laboratory Reproducibility

The total fluorine concentrations measured on paper packaging substrates using the F-ISE method described were tested for reproducibility with an external laboratory result [23]. Results show that, using the same paper packaging substrates and following the same F-ISE methodology utilizing a different ISE probe, meter, and grinder, there is no statistically significant difference (*p* > 0.05) between the results from the two labs (Table 7).

## 5. Conclusions

The key to successfully implementing the protocol on determining total fluorine and organic fluorine using the ion selective electrode method will require the continuous validation of the step-by-step process. The ISE method does not require sophisticated analytical equipment; however, proper personnel training is required in following the protocols consistently, including the critical steps involved. The robustness of this method as a screening test for determining total fluorine and total organic fluorine in food packaging was demonstrated by an external laboratory with similar results using different equipment.

The efficiency of this F-ISE method and its safety relies on the following considerations:

- One aspect that needs to be emphasized is the sample preparation protocol, which is also the time-consuming portion of the method and can dramatically influence the results. It is to be noted that different packaging substrates (paper vs. plastic) will vary in the amount of time required to achieve the desired size to pass through a 425 μm sieve (plastic often takes more time to mill) and mass to fill a gelatin capsule. Moreover, complete combustion will give more accurate results, provided all combustion products are collected and properly handled.
- Cleaning between each sample with solvent and materials known to have low fluoride concentrations is extremely important to prevent contamination. Laboratory tools used in milling, screening, and combustion should be rinsed with methanol after washing to remove organic fluorine/fluoride contamination. Additionally, it is important to rinse the ISE probe, temperature probe, and stirrer with DI water between each sample and gently blot dry with a lint-free wipe. Lastly, all reusable storage containers and glassware must be scrubbed thoroughly with soapy water and rinsed with methanol, while counters and equipment need to be wiped down regularly to remove sample debris.
- Moreover, the regular calibration and validation of the ISE probe is critical in sample analysis. The calibration and validation of the ISE probe must occur every day before measuring samples for total fluorine and organic fluorine.
- Wearing proper protective equipment while conducting the test assures the safety of all the personnel involved in testing.
- Consistency is the key to accurate and reproducible results. Following the protocol step by step will ensure accurate and repeatable results.
- Practice and diligent research record-keeping is important in tracking errors, refining procedures, and validating results.

## Figures and Tables

**Table 1:** Guide to common testing methods for total fluorine and total organic fluorine.

Analytical Techniques	Applications	Target Substance(s)	Additional Information
Combustion Ion Chromatography (CIC)	Food packaging Firefighting foams Water samples Most organic solids	Total fluorine or total organic fluorine	Offers possibility of fast, accurate, high sensitivity analysis Destructive to sample Total fluorine of the entire sample, independent of original material thickness, is measured by integrated direct combustion followed by chromatographic separation and conductivity detection. Combined with a separate measurement of a sample subjected to water extraction of inorganic fluorine prior to combustion total, organic, and inorganic fluorine can be measured. Alternatively, the organic fluorine can be either extracted or adsorbed and combusted independent of original matrix to measure total organic fluorine (either extractable or adsorbable). These techniques result in lower limits of reporting than direct combustion through various methods to concentrate fluorine containing materials for measurement. These techniques are known as extractable organic fluorine (EOF) and adsorbable organic fluorine (used for waters/wastewater) methods. Technique referenced in Clean Production Action’s firefighting foam standard—1 ppm total organic fluorine threshold requirement for certification Commercially available and expensive
Instrumental Neutron Activation Analysis (INAA)	Food packaging Textiles Other organic materials	Total fluorine	Measures total of entire sample, independent of thickness Non-destructive and rapid Since technique relies on nuclear, rather than chemical reaction, samples may be analyzed without dissolution or decomposition No chemical preparation required Samples are irradiated, followed by a decay period, emitting gamma rays, and target nuclide identified via gamma ray spectroscopy. Quantification accomplished by comparison with standards. Not commercially available
Oxygen Combustion and Ion-Specific Electrode (ISE)	Food packaging Most organic solids	Total fluorine or total organic fluorine, depending on sample preparation	Combustion in oxygen atmosphere with known amount of buffer solution; volumetric dilution with ionic buffer solution, then analyzed with fluoride ion selective electrode Destructive method, low-cost, and commercially available
Particle-Induced Gamma Emission (PIGE)	Food packaging Firefighting foam	Total fluorine	Surface measurement, so results dependent on sample thickness Good result accuracy, well-used, cost-effective sample analysis Not commercially available, expensive equipment Advantage to probe surfaces
Quadruple Time-Of-Flight-Mass Spectrometry (QTOF-MS)	Water samples Materials	Full range of potential compounds in the PFAS family	QTOF-MS combines time of flight and quadrupole instruments, a pairing that results in high mass accuracy; speed and sensitivity are benefits of the QTOF Coupled with LC or GC for analyte determination Expensive and time consuming Other tandem mass spectrometry instruments available, such as triple quadrupole and orbitrap (ms^n^)
Total Oxidizable Precursors (TOP) Assay	Foam products Textiles Water samples	Quantifies total amount of chemical precursors to perfluoroalkyl acids (PFAAs)	Selective PFAS method (only those that can be oxidized to form targeted PFAAs); destructive, relatively rapid, and low cost Sample treated so precursor substances contained within the sample are oxidized, then PFAS determination using methods such as LC-MS/MS

**Table 2:** Dilutions for standards preparation.

Stock Used	Final ppm	Fluoride Stock Volume (mL)	ACS Water Volume (mL)	TISAB II Volume (mL)	Total Volume (mL)
1000 ppm	1000 ppm	5	0	5	10
1000 ppm	500 ppm	2.5	2.5	5	10
100 ppm	100 ppm	5	0	5	10
100 ppm	50 ppm	2.5	2.5	5	10
100 ppm	25 ppm	1.25	3.75	5	10
10 ppm	10 ppm	5	0	5	10
10 ppm	1 ppm	0.5	4.5	5	10
10 ppm	0.1 ppm	0.05	4.95	5	10

**Table 3:** Recommended table for sample preparation for total fluorine analysis. E.g. prepared sample data.

Sample No.	Sample Description	Combustion Aid (g)	Sample Weight (g)	Notes
Example Sample 1	Paper	Starch, 0.0000	0.0000	Complete/incomplete combustion

**Table 4:** Recommended layout of calibration curve ad sample values for reference. E.g., processed data results.

Sample No.	Sample Description	mV Measured	Sample Weight (kg)	Slope:	Y-Int:	Using slope, x=	ppm (µg/mL)	Sample Volume (mL)	ppm (mg/kg)
Example Sample 1	Paper	143.7	0.0001676	−26.02	106.1	−1.446	0.236	50.00	70

**Table 5:** Total fluorine measurements for repeatability evaluation.

Sample	Replicate	Measured Total Fluorine (ppm)	Mean	Standard Deviation	%CV
A	1	13.36	13.54	0.91	7.0
	2	12.60			
	3	15.06			
	4	13.35			
	5	13.34			
B	1	172.91	167.84	12.29	7.0
	2	153.08			
	3	158.54			
	4	184.19			
	5	170.47			
C	1	16.71	17.20	1.48	9.0
	2	19.56			
	3	16.68			
	4	15.60			
	5	17.47			

**Table 6:** Total fluorine pre-extraction spike recovery data.

Sample	Replicate	Theoretical Spike Concentration (μg/mL)	Measured Value (μg/mL)	Observed Spike Concentration (μg/mL)	Spike Recovery (%)
Unspiked	1	-	8.55	-	-
	2	-	6.61	-	-
Near LOD spike	1	12.5	17.46	9.88	79.04
	2	12.5	19.82	12.24	97.92
Near LOQ spike	1	37.5	46.94	39.86	104.95
	2	37.5	49.6	42.01	112.03
50% high standard spike	1	375	395.4	387.82	103.42
	2	375	385.87	378.29	100.88
120% high standard spike	1	900	932.52	924.94	102.77
	2	900	905.89	898.3	99.81

**Table 7:** Inter-laboratory reproducibility results *.

Sample	External Lab	Iowa State University Lab
1	20	14
2	20	48
3	17	24
4	357	385
5	402	371
6	370	521
7	372	496

* The paired *t*-test produced a *p* (T ≤ t) two-tail value greater than 0.05.

## Data Availability

The data presented in this study are available on request from the corresponding author.
